# Characterization of Lumpy Skin Disease in Northern Bangladesh: Clinical, Pathological, Biochemical, and Molecular Perspectives

**DOI:** 10.1155/vmi/4623554

**Published:** 2025-07-21

**Authors:** Samiron Roy, Mahfuza Akther, Md. Sadequl Islam, Mirza Mienur Meher, Sumon Sarkar, Md. Shakil Islam, Jahagir Alam, Md. Mominul Islam

**Affiliations:** ^1^Department of Pathology and Parasitology, Hajee Mohammad Danesh Science and Technology University, Dinajpue 5200, Bangladesh; ^2^Department of Anatomy and Histology, Hajee Mohammad Danesh Science and Technology University, Dinajpur 5200, Bangladesh; ^3^Department of Microbiology and Public Health, Faculty of Veterinary Medicine and Animal Science, Gazipur Agricultural University, Gazipur 1706, Bangladesh; ^4^Department of Physiology and Pharmacology, Hajee Mohammad Danesh Science and Technology University, Dinajpur 5200, Bangladesh; ^5^Department of Pharmacology and Toxicology, Sher-e-Bangla Agricultural University, Dhaka 1207, Bangladesh; ^6^Department of Anatomy, Histology and Physiology, Sher-e-Bangla Agricultural University, Dhaka 1207, Bangladesh; ^7^Deparment of Pathology, Sher-e-Bangla Agricultural University, Dhaka 1207, Bangladesh

**Keywords:** clinical signs, gross pathological, histological, phylogenic tree, serum biochemical

## Abstract

Lumpy skin disease (LSD) is an economically important disease of cattle, considered as a threat to the livestock industry. This study aimed to assess the clinical symptoms, gross pathology and histopathology, serum biochemical values, and molecular characterization of LSD in cattle. Cattle in Dinajpur, Thakurgaon, Nilphamari, and Rangpur affected by LSD were initially diagnosed through clinical signs and gross lesions. Blood samples were then collected for biochemical analysis and molecular detection through PCR, and sequencing was performed to characterize the LSD virus (LSDV). The prevalence of LSD in northern areas of Bangladesh was 33.44%. The recorded clinical signs were high fever; firm, raised skin nodules around the head, neck, and limbs; swelling of the limbs and brisket area; and rough hair coat, nasal discharge, dyspnea, corneal opacity, and severe weakness. Grossly, a well-defined, hard swelling of around 1–3.00 cm in diameter in the skin accompanied enlargement of the nearby lymph nodes, and the nodules are composed of a hard, creamy-gray, or yellow clump of the tissue. Histopathologically, ballooning degeneration of the epidermal cell layers, intracytoplasmic eosinophilic inclusion bodies in distinct dermal and epidermal cells, necrosis with substantial infiltration of inflammatory cells, and congested blood vessels in the dermal layer were noted. While total protein, BUN, serum AST, and ALT were increased significantly (*p* < 0.05), the serum creatinine, GGT, and total albumin were not changed significantly compared with the healthy cattle. Phylogenetic analysis showed that the circulating isolates from northern part of Bangladesh were found in the same clades with India, Pakistan, and Thailand. Thus, this study provides crucial findings on this emerging disease, including its gross and histopathology, serum biochemical properties, and molecular epidemiology in the northern regions of Bangladesh.

## 1. Introduction

Lumpy skin disease (LSD) is a vector-borne severe viral disease [1], particularly in cattle that has emerged as a major concern in the livestock industry, being primarily transmitted by mosquitoes [2], flies [3], and ticks [4]. This disease is caused by a virus belonging to the genus Capri poxvirus of the family Poxviridae [5]. The disease is characterized by the formation of firm, circumscribed, and raised skin nodules, which can range in size from 1 to 3 cm in diameter and 1 to 2 cm in depth [1]. The other clinical signs are anorexia, depression, ocular and nasal discharges, and edematous swellings [6]. The morbidity and mortality rate of LSD virus (LSDV)-infected animals varies, mainly based on the immunity of the host and management practices in the farm [7]. Epidemiological studies have revealed that the disease has a significant impact on the livelihoods of farmers, with overall economic losses ranging from US$9.6 to US$6340 per affected farm, depending on the production system and species affected [8]. This economic loss occurs in the form of reduced body weight, decreased milk production, degradation of hide quality, abortion and sterility, and death of the animal [9]. LSDV has long been known for its endemic presence in Africa [10]. In the last decade, the virus spread to the Middle East and European countries. In Southeast Asia, the virus was first recorded in 2019 in Bangladesh, Nepal, India, Myanmar, Thailand, and China [11]. The emergence of disease in noninfected countries is increasing throughout the world, emphasizing the need for a better understanding of the biology of the virus and the virulent factors of the disease.

Diagnosis of LSD is always challenging, especially in low- to middle-income countries. To put effective control measures in place and prevent the spread of this economically significant disease, an accurate and prompt diagnosis of LSD is essential. The diagnosis of LSD is typically based on the combination of clinical features, gross and histopathological lesions and molecular confirmation. Gross lesions include characteristic nodular lesion which may be firm and show central necrosis with sit fast appearances, swollen lymph nodes, subcutaneous edema and hemorrhage in the internal organs also noticed [12, 13]. In addition, ballooning degeneration, intracytoplasmic eosinophilic inclusion bodies, and hyperplastic hair follicles in histopathology also supplemented in diagnosis of LSD [14]. Molecular methods, particularly polymerase chain reaction (PCR)-based detection methods can be useful for rapid and accurate diagnosis of the disease [15]. A thorough clinicopathological examination consisting of clinical assessment, gross lesions, histopathology, and correlation with laboratory findings is essential for the diagnosis of disease. The effective control and prevention of LSD are based on accurate surveillance methods for rapid detection of disease outbreak and their management and routine vaccination program [16].

LSDV is antigenically closely related and indistinguishable from other Capri pox viruses (CaPVs) such as goat pox virus and sheep pox virus [17]. Therefore, accurately distinguishing between different strains of Capri poxviruses like LSDV is crucial for effective management and control of LSD. Several gene-based sequencing methods targeting specific genes like *RPO30, GPCR, B22R*, and *EEV* glycoprotein have been used to differentiate CaPVs [18]. The antigenic resemblance with countries nearby such as India, Nepal, Myanmar, and Thailand, as well as some with Kenya, is revealed by phylogenetic analysis of LSDV collected from central and southern areas in Bangladesh [6, 13, 19]. Despite the high-level similarity between strains, the severity of LSD in Bangladesh is higher than in other parts of the world with high mortality [20]. However, no molecular and pathological studies have been conducted in the northern area of Bangladesh. Hence, the present study focused on the pathological, biochemical, and molecular characterization of LSDV collected from the northern areas of Bangladesh.

## 2. Materials and Methods

### 2.1. Study Design

The research was conducted from July 2022 to June 2023 across selected upazilas in the Dinajpur, Thakurgaon, Nilphamari, and Rangpur districts of Bangladesh. A total of 2880 cattle were randomly selected for the study from these districts, with 725 cattle from Dinajpur, 705 from Thakurgaon, 690 from Nilphamari, and 760 from Rangpur. The selection of areas and cattle was guided by reports from local registered veterinarians followed by farm owner's concerns and complain. Related data were collected and gathered by on-spot visit to the farms. Each cattle served as the sampling unit, and a positive case of LSD was defined by nodular lesions of varying sizes on their skin, accompanied by fever, lameness, swollen lymph nodes, joint edema, and reduced productivity like decreased milk yield and abortion [21]. All the selected cattle were in different age groups and composed of different breeds including local, exotic, and cross. Samples for further analysis were collected from those animals which exhibit febrile and/or nodular lesions in the skin. In total, 33 skin biopsy samples, 33 blood samples in EDTA vacutainers, 15 nasal swabs, and 15 fecal swabs were collected randomly from infected cattle using simple random sampling methods.

### 2.2. Gross and Histopathological Examination

According to Kashem et al.'s guidelines, skin biopsy samples were aseptically taken from nodular lesions [22]. The biopsy sites were shaved with sterile blades, and small punches were made to collect tissue from the skin and subcutaneous layers. Half of each biopsy sample was preserved in 10% neutral buffered formalin for histopathological examination using conventional hematoxylin and eosin (H&E) staining [23], while the remaining half was stored in phosphate-buffered saline (PBS) at −20°C for molecular analysis.

### 2.3. Biochemical Examination

Blood samples (5 mL) were drawn from the jugular veins of each animal using vacutainer tubes containing EDTA, using atraumatic jugular venipuncture. Blood was collected from six LSDV-infected animals and three noninfected animals as a control. The six infected animals were selected based on the clear and typical clinical signs, and availability and cooperation of animal owner during sampling. The three control animals were clinically healthy, unvaccinated against LSDV, and from the same or nearby farms with no history or signs of LSD at the time of sampling. These samples were then sent to the laboratory for biochemical analysis using an automated analyzer (ILab-300 Biomerieux Diagnostic, Milan, Italy). Various biochemical parameters, including albumin, serum creatinine, serum cholesterol, aspartate aminotransferase (AST), alanine aminotransferase (ALT), alkaline phosphatase (ALP), total protein (TP), gamma-glutamyl transferase (GGT), and blood urea nitrogen (BUN), were measured.

### 2.4. DNA Extraction and PCR for LSDV Detection

Skin biopsy samples were homogenized using a mortar and pestle and transferred to sterile tubes containing 10 mL of PBS. After centrifugation at 1000 g for 10 min, 200 μL of supernatant was transferred to an Eppendorf tube for DNA extraction. Genomic DNA was isolated from skin samples, blood, nasal swabs, and fecal swabs using a Qiagen mini-DNA extraction kit following the manufacturer's instructions, with minor modifications. PCR was performed to detect the presence of LSDV using primers targeting the P32 gene (forward: AAGTTACTTATATGGGAAAAGG, reverse: GTGTTATCATCTTCTATAACAAC) [24]. The PCRs were conducted on a K960 Gradient thermocycler (Biorad, USA) with the following thermal conditions: 94°C for 2 min, followed by 40 cycles of 94°C for 20 s, 55°C for 10 s, and 72°C for 20 s, ending with a 5-min extension at 72°C. The PCR products were then analyzed by 1.5% agarose gel electrophoresis staining with ethidium bromide and visualized under UV light. The expected amplicon size was approximately 203 base pairs.

### 2.5. Phylogenetic Tree Analysis

GPCR [25] and P32 [26] genes of four LSDV isolates were amplified and sequenced according to previous published protocols. PCR amplification was performed in a 25 μL reaction mixture comprising 1 μL of each forward and reverse primer, 12.5 μL of 2X green Mastermix (Promega, USA), 5 μL of template DNA, and nuclease-free water to reach the final volume of 25 μL. The thermal cycling conditions included an initial denaturation at 95°C for 4 min, followed by 35 cycles of denaturation at 95°C for 30 s, annealing at 56°C for 30 s, and extension at 72°C for 45 s. A final extension step was carried out at 72°C for 7 min. PCR amplicons were visualized by electrophoresis on a 1.5% agarose gel run at 100 V for 1 h. Sequencing of the partial GPCR and P32 genes was performed in the National Institute of Biotechnology, Dhaka, Bangladesh. The quality of the P32 and GPCR gene sequences was checked, trimmed, and aligned using BioEdit software (https://bioedit.software.informer.com/). The similarity of the sequences was observed with other P32 and GPCR sequences using the NCBI BLAST program. Then, the phylogenetic tree was analyzed using MEGA 11 software (https://www.megasoftware.net). The phylogenetic tree of P32 and GPCR gene was created using the maximum likelihood method with 1000 generations. The sequences of the P32 gene were submitted to the GenBank database and are available under accession numbers PQ584629 to PQ584632. The sequences of the GPCR gene were submitted to the GenBank database and are available under accession numbers PQ585742 to PQ585745.

### 2.6. Statistical Analysis

All data were compiled and coded using Microsoft Excel 2016, and the results were expressed as mean standard deviations. The two-sample *t*-test was employed to assess the differences between LSDV-infected and healthy cattle. A *p* value of less than 0.05 was considered statistically significant. Statistical analysis was performed using SPSS version 25.0 (SPSS, Inc., Chicago, IL, USA).

## 3. Results

### 3.1. Clinical Findings on Field Investigation

This study investigated 2880 cattle from the northern region (Dinajpur, Thakurgaon, Nilphamari, and Rangpur districts) of Bangladesh. Among them, 963 cattle (33.44%) exhibited typical clinical signs of LSD. The highest prevalence was recorded as 36.74% in Thakurgaon, followed by Rangpur (34.47%), Dinajpur (32.97%), and Nilphamari (29.42), respectively. In addition, 251 animals (8.71%) were dead due to the infection of LSD in cattle (Table 1), with the highest mortality rate detected in Thakurgaon (10.05%).

**Figure 1:**
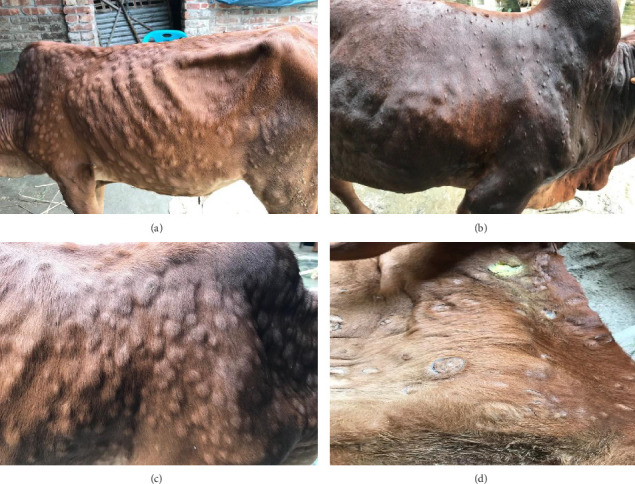
Clinical presentation of LSD in cattle. (a) Nodule formation in the whole body of the cattle. (b) Nodules are smaller in size in the early days of infection. (c) Nodules are circumscribed, firm, raised areas with 1–3 cm in diameter. (d) Nodules are necrosed, heading to wounds and occasionally sloughing off.

The major clinical signs in infected cattle were generalized skin nodules of various size observed all over the body (Figure 1(a)) along with fever, hypersalivation, anorexia, nasal and lacrimal discharge, and edema in the limb and brisket region. In the first 2–3 days, the skin nodules appeared as minute raised skin (Figure 1(b)), and later, nodules developed gradually 2–3 cm in diameter in 7–10 days (Figure 1(c)). Subsequently, after 2 weeks of their appearance, nodules became ruptured, necrotic, and ulcerated (Figure 1(d)). In addition, necrotic plaques in the eyes were also present in some animals mainly in young calves.

### 3.2. Pathological Findings

**Figure 2:**
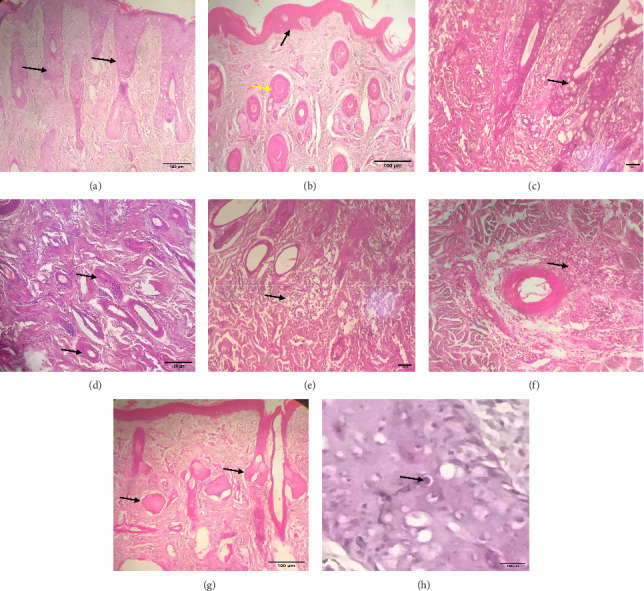
Histopathological lesions of LSDV-affected cattle skin (H&E stain). (a) Acanthosis and vacuolation of the epidermis; (b) epidermal and follicular hyperplasia; (c) ballooning degeneration of the keratinocytes; (d, e) mononuclear cell infiltration in the dermis; (f) vasculitis with perivascular mononuclear cell infiltration; (g) dilation of the sebaceous gland; (h) intracytoplasmic eosinophilic inclusion bodies.

Grossly, well-developed, circumscribed, and firm nodules were found all over the body. The nodules were composed of a gray or yellowish mass of tissues. Swellings of the limb and brisket area were also found. Moreover, enlargement of the regional lymph node was also found in LSD-infected cattle. Histopathology of the skin nodules revealed hyperkeratosis and acanthosis of epidermis (Figure 2(a)), thickening of epidermis and follicular hyperplasia with surrounding mononuclear cell infiltration (Figure 2(b)), ballooning degeneration of the keratinocytes (Figure 2(c)), varying degrees of mononuclear cell infiltration with vasculitis (Figures 2(d), 2(e), 2(f)), dilatation of sweat and sebaceous glands with edema in the dermis (Figure 2(g)), and occasional formation of intracytoplasmic eosinophilic inclusion bodies in the epithelial cell (Figure 2(h)).

### 3.3. Biochemical Alteration

We observed significant differences in biochemical parameters between LSD-infected and healthy cattle. Albumin, serum creatinine, and GGT values were not significantly increased in LSD-infected cattle in comparison to healthy animals (Table 2). On the other hand, the biochemical values of SGOT, SGPT, and serum cholesterol were significantly higher in LSD-infected animals than the healthy cattle (*p* > 0.05) (Table 2). In addition, this study also found that serum ALP, BUN, and TP levels were markedly increased in LSD-infected cattle (*p* > 0.005) (Table 2).

### 3.4. Molecular Findings and Phylogenetic Explanation

**Figure 3:**
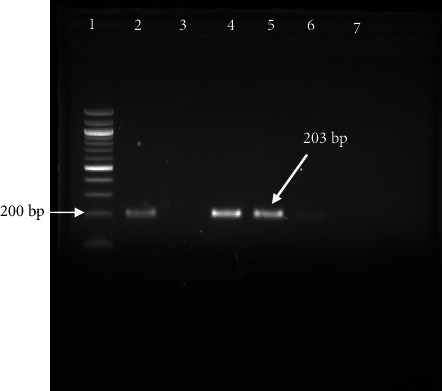
PCR amplification of lumpy skin disease virus. Well 1 contains a DNA ladder, Wells 2–6 contain different samples, and Well 7 contains negative control. LSDV DNA is shown at 203 bp by using a 100-bp DNA ladder.

The confirmation of LSDV infection was done by PCR amplification of the P32 gene. Gel electrophoresis of the P32 gene at 203 bp confirms the presence of LSDV in cattle which exhibited the clinical signs of LSD infection (Figure 3). While all skin nodule samples (100%) tested positive by PCR, molecular confirmation from blood, nasal swab, and fecal swab samples was achieved in 8 (24.24%), 3 (20%), and 0 (0%) cases, respectively.

**Figure 4:**
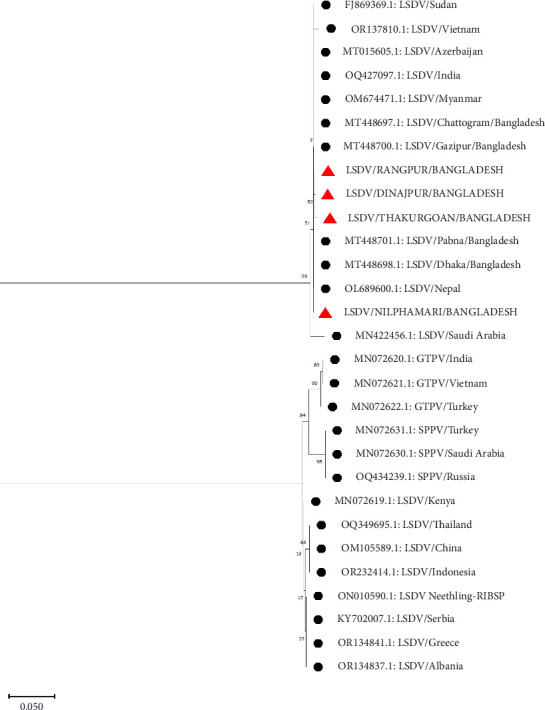
Phylogenetic analysis of GPCR gene sequences obtained from northern Bangladesh (red triangle) with other Capri pox viruses.

**Figure 5:**
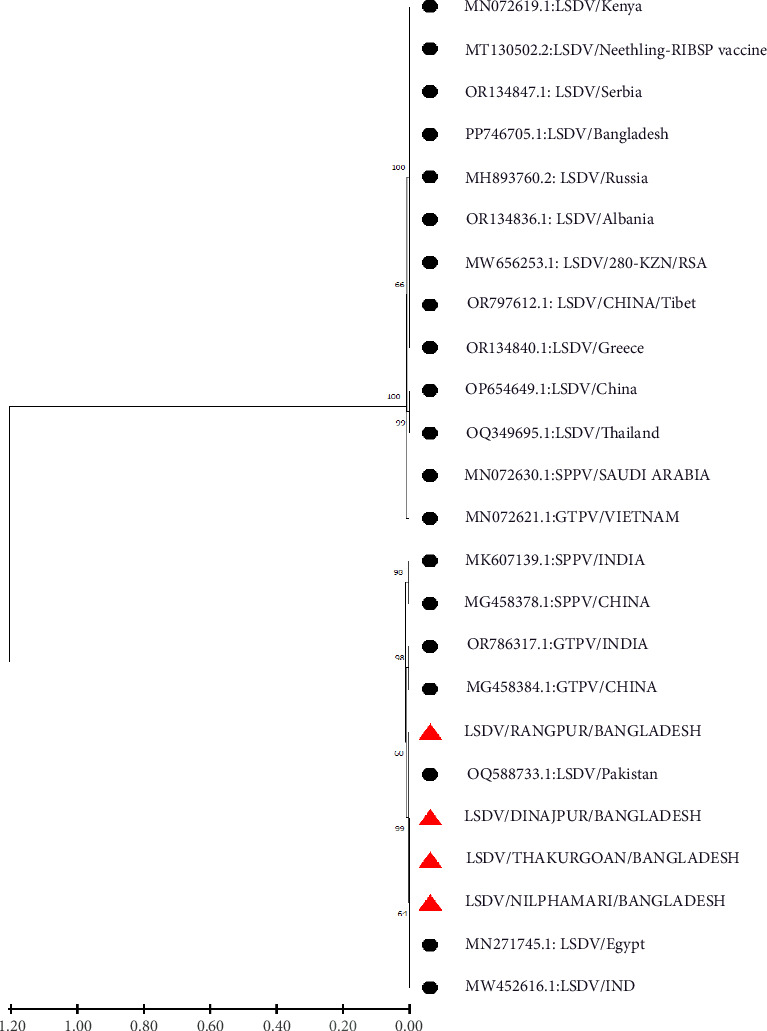
Phylogenetic analysis of P32 gene sequences obtained from northern Bangladesh (red triangle) with other Capri pox viruses.

P32 and GPCR genes were successfully amplified and sequenced from four LSDV-positive samples. The sequence of GPCR genes from this study was 99.5%–100% identical with each other. NCBI BLAST results showed 99%–100% similarity of the nucleotide sequences with the isolates of Bangladesh and other neighboring countries. The phylogenetic analysis of the GPCR gene revealed that isolates from this study clustered tightly with another Bangladesh isolates from previous studies, and isolates from Nepal, India, Pakistan, and Vietnam were also found in the same clade. However, isolates from Africa, Russia, Egypt, China, and Serbia are segregated into different clades (Figure 4). In addition, phylogenetic analysis of the P32 gene demonstrated that the isolates from this study were closely related to isolates from Pakistan, India, and Egypt (Figure 5).

## 4. Discussion

LSD is an emerging transboundary disease, and it was restricted to Africa for a longer period, but it has recently spread out in different parts of the world including Bangladesh [11]. The disease was reported all over Bangladesh and was responsible for great economic loss for the cattle industry of this country. Understanding the prevalence and characteristics of the disease is crucial for developing effective control measures, and clinicopathological studies consider an important investigation in the diagnosis and understanding of diseases. The present study aimed to investigate the prevalence, clinical manifestations, pathological, and molecular characteristics of LSDV-affected cattle in the northern region of Bangladesh. The samples were collected from the four different districts with a span of 200 km. This study noted 33.44% prevalence of LSD in northern Bangladesh, with a mortality of 8.71%. These findings are consistent with the reports in northeast Bangladesh, where prevalence rates have ranged from 35% to 41% depending on the breed and age of the cattle [11, 27]. However, a higher prevalence rate (71%) was recorded in other regions of Bangladesh with a 7.14% mortality [28]. Other authors recorded a wide range frequency of LSD globally, ranging from 7% to 35% [29–31]. The higher prevalence of LSD in Bangladesh may be due to the easy movement of the animal and the availability of the vectors that facilitate the disease transmission. In Bangladesh, most of the farms are small and trading and transfer of animals between farms are a frequent event. This may increase the risk of spreading the virus between farms, subsequently higher occurrences of the disease in these regions. In addition, the differences in disease occurrence compared to some other studies might be due to differences in herd immunity, management practices, or environmental factors [32]. However, the variation in prevalence and mortality underscores the need for region-specific interventions, including targeted vaccination campaigns and improved surveillance to control the spread of LSD.

While previous reports have documented LSD in Bangladesh, there is a lack of comprehensive research on the clinical manifestations, histopathological changes, and biochemical profiles in affected cattle [27, 33]. Clinically, the study found that LSD-infected cattle exhibited a range of symptoms, including fever, hypersalivation, anorexia, edema in the limb and brisket areas, and generalized skin nodules. These clinical signs are characteristic of LSD and have been widely documented in the literature [7, 34, 35]. The progression of skin nodules from small, raised areas to large necrotic ulcers over 7–14 days is a hallmark of the disease, reflecting the virus's ability to induce a strong inflammatory response and tissue necrosis. The occurrence of necrotic plaques in the eyes of young calves, although less commonly reported, aligns with the systemic nature of the disease and its potential to affect multiple organ systems [36]. The pathological findings in this study provide further evidence of the severe impact of LSD on cattle. Grossly, the presence of well-developed, circumscribed, and firm nodules composed of gray or yellowish masses of tissue were noted, alongside swelling of the limb and brisket areas and enlargement of regional lymph nodes. These findings are consistent with other studies that have documented similar gross lesions in LSD-infected cattle [27]. Microscopically, the observation of ballooning degeneration of epidermal cells, intracytoplasmic eosinophilic inclusion bodies, and necrosis with heavy infiltration of inflammatory cells highlights the extensive tissue damage and immune response triggered by the virus, as reported in a previous study [6]. The presence of acanthosis, parakeratosis, and hyperkeratosis in the epidermis further supports the chronic inflammatory nature of LSD, as these changes are typically associated with prolonged or severe skin infections [37]. Biochemically, significant differences were observed between LSD-infected and healthy cattle. The study found that SGOT (AST), SGPT (ALT), and serum cholesterol levels were significantly higher in LSD-infected cattle, indicating liver damage or stress. These findings align with other research showing that LSD can induce hepatocellular injury, likely due to the systemic inflammatory response and direct viral effects on the liver [38]. The marked increase in serum ALP, BUN, and TP levels further suggests liver involvement and a possible impact on renal function or protein metabolism. The stability of albumin, serum creatinine, and GGT levels suggests that while there is significant liver and systemic involvement, these functions may remain relatively preserved in many cases unless the disease is particularly severe [6, 39].

Clinical samples from skin nodules were taken for molecular confirmation through PCR. All the samples were found positive in our study and under field diagnosis based on the striking clinical symptoms of LSD-affected cattle. This finding agrees with the previous study which recorded almost 100% positivity of LSDV collected from skin lesions [6, 39] due to the high number of viral loads in the skin nodules. Our molecular and phylogenetic findings on the LSDV provide important insights into the genetic relationships and potential transmission routes of the virus within and beyond South Asia. The successful amplification and sequencing of the GPCR gene from LSDV-positive samples, with the sequences showing 100% identity, suggests a high level of genetic stability among these isolates. The genetic homogeneity observed in the study could suggest recent common ancestry or limited viral evolution within the region. This is consistent with other studies in South Asia, where LSDV strains have shown limited variation, likely due to the virus's relatively recent introduction and spread in this region [19, 40]. The phylogenetic analysis where isolates from Bangladesh clustered closely with those from neighboring countries such as Nepal, India, Pakistan, and Vietnam further underscores the regional spread of LSDV. This clustering supports the hypothesis of a regional transmission network facilitated by cross-border livestock movements and trade, a well-documented factor in the spread of livestock diseases in South Asia [41]. In contrast, the segregation of the isolates from those found in Africa, Russia, Egypt, China, and Serbia highlights the genetic divergence of LSDV strains on a global scale. This divergence likely reflects the historical spread of LSDV from its origins in Africa to other parts of the world, including Europe and Asia. African LSDV strains, which are genetically distinct, are considered ancestral, with subsequent diversification occurring as the virus spreads to new regions [7]. The global genetic diversity observed in LSDV strains can be attributed to different introduction events, varying selective pressures, and the virus's adaptation to different ecological and agricultural environments [42].

## 5. Conclusion

This study represents the first comprehensive investigation into the clinico-pathological and molecular characteristics of LSDV in naturally infected cattle within the northern regions of Bangladesh. The findings, including the presence of distinct clinical signs associated with LSD, positive PCR results, and characteristic histopathological changes in tissue samples, provide strong evidence that LSDV is widely circulating among cattle in these areas. These results underscore the urgent need for a detailed molecular characterization of LSDV isolates specific to the affected districts. Such analysis would facilitate the development of a strain-specific vaccine, which is essential for effective disease control. Implementing this strategy would not only safeguard animal health but also improve livestock productivity and ensure the sustainability of cattle farming in the region.

## Figures and Tables

**Table 1:** LSD prevalence of cattle in different districts of northern Bangladesh.

Location	Number of cattle on the farms	Number of affected cattle (%)	Number of deaths (%)	Case fatality rate (%)
Dinajpur	725	239 (32.97)	60 (8.27)	25.10
Thakurgaon	705	259 (36.74)	71 (10.05)	27.41
Nilphamari	690	203 (29.42)	58 (8.40)	28.57
Rangpur	760	262 (34.47)	62 (8.15)	23.66
Total	2880	963 (33.44)	251 (8.72)	26.06

**Table 2:** Biochemical alteration in LSD-affected cattle.

Biochemical test	Non-LSD (*n* = 3)	LSD (*n* = 6)	Level of significance
Albumin (g/dL)	4.23 ± 0.15	4.45 ± 0.24	NS
S. creatinine (mg/dL)	0.57 ± 0.03	0.77 ± 0.07	NS
S. cholesterol (mg/dL)	116.9 ± 1.92	150.48 ± 9.22	^∗^
SGPT (ALT) (U.l)	20.5 ± 0.96	38 ± 5.96	^∗^
SGOT (AST) (U.l)	18.63 ± 0.73	46 ± 7.47	^∗^
S. alkaline phosphatase (U.l)	32.97 ± 0.45	90.67 ± 8.84	^∗∗^
BUN (mg/dL)	9.7 ± 0.6	16.28 ± 1.23	^∗∗^
GGT (U.l)	22.07 ± 2.1	27.07 ± 3.72	NS
Total protein (TP) (g/dL)	6.33 ± 0.13	9.72 ± 0.38	^∗∗^

*Note:* S. denotes serum.

^∗^denotes statistically significant at *p* < 0.05.

^∗∗^denotes statistically significant at *p* < 0.005.

## Data Availability

The data that support the findings of this study are openly available in GenBank at https://www.ncbi.nlm.nih.gov, reference numbers PQ585742 to PQ585745.
